# Improving malaria control by understanding human behaviour

**DOI:** 10.2471/BLT.20.285369

**Published:** 2021-09-30

**Authors:** April Monroe, Bolanle Olapeju, Sarah Moore, Gabrielle Hunter, Alice Payne Merritt, Fredros Okumu, Stella Babalola

**Affiliations:** a Johns Hopkins Center for Communication Programs, Suite 310, 111 Market Place, Baltimore, Maryland 21202, United States of America.; b Environmental Health and Ecological Sciences Department, Ifakara Health Institute, Ifakara, United Republic of Tanzania.

Malaria, a preventable and treatable mosquito-borne disease, kills an estimated 409 000 people each year.^1^ Tremendous progress has been made in the fight against malaria thanks to the large-scale roll out of effective prevention, testing and treatment interventions; however, despite these gains, progress has recently begun to plateau.^1^ While many factors contribute to this stalling, one important and often overlooked factor is how individuals, families and communities take actions and adopt habits to protect themselves from malaria. Here we discuss the importance of human behaviour for malaria control programmes and provide an example of how behaviour change theory can be used to inform evidence-based social and behaviour change interventions.

## Human behaviour and malaria

Malaria prevention efforts depend heavily on behaviours occurring at multiple levels including at the individual, household, community and societal levels. Households need to obtain insecticide-treated nets, a frontline malaria control intervention, through available distribution channels (national malaria control programmes and partners typically provide these). Furthermore, individuals must use the nets consistently and care for them appropriately. Indoor residual spraying, another core malaria intervention, depends on the deployment of well-trained spray teams and people accepting these teams in their homes, agreeing to remove their possessions from the house before spraying and refraining from major post-spray modifications of walls such as painting or hanging decorations, which can reduce spray efficacy. Supplementary vector control tools can help fill gaps in protection that nets and spraying are not able to fill, such as outdoor exposure to malaria mosquitoes. Examples of promising approaches include spatial repellents, attractive targeted sugar baits, endectocides, larval source management and housing modification. However, no matter how efficacious an approach is, it will only be effective if people engage with it and use it appropriately.

Human behaviour also plays an important role in case management for malaria. Individuals, and especially caretakers of young children, must promptly seek care for fever from appropriate health facilities and complete the treatment course as prescribed. Likewise, pregnant women must attend antenatal clinics on schedule to receive and complete the recommended doses of preventive treatment. Health-care providers must adhere to national clinical guidelines on malaria testing and treatment, and prevention of malaria in pregnancy; accurately complete clinical registries and reports; manage stocks of commodities in health facilities; and consistently demonstrate technical competence and interpersonal communication skills.

## Drivers of human behaviour

**Fig. 1:**
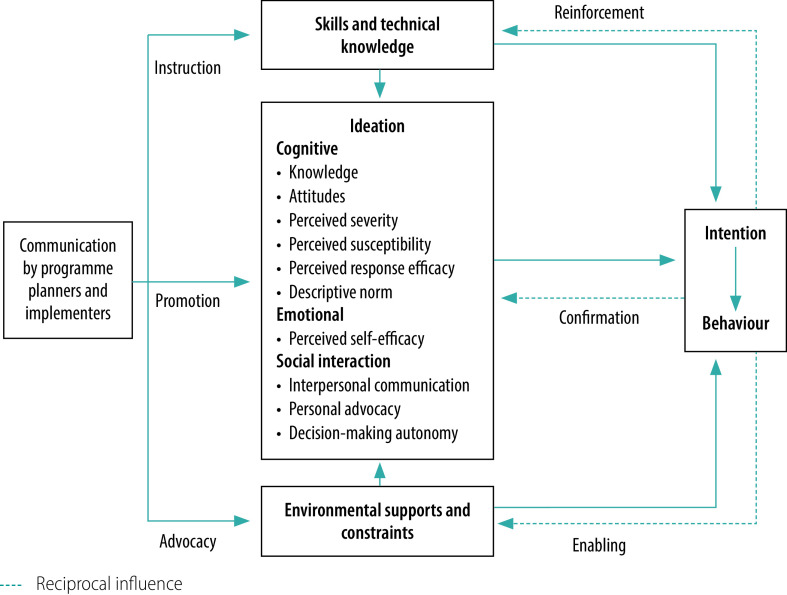
Ideation model of strategic communication and behaviour change

Note: The model shows the pathway between social and behaviour change interventions, psychosocial and environmental factors, and behaviour change.

Predictive models of behaviour change can play an important role in informing effective social and behaviour change interventions. Ideation, a meta-theoretical model of behaviour change, has been used to understand, predict and influence a range of health behaviours, first concerning family planning^2^^–^^5^ and recently malaria.^6^^–^^11^ Ideation refers to a cluster of interrelated psychosocial variables that characterize how someone thinks and feels about a particular behaviour and how new ways of thinking diffuse through a population via communication and social interaction.^2^ The ideation model (Fig. 1) draws on cognitive, emotional and social constructs from multiple behavioural theories, including the Health Belief Model, the Theory of Planned Behaviour, the Extended Parallel Process Model and the Social Cognitive Theory.

Data source: Adapted from Kincaid, 2000.^2^

The underlying premise of the ideation model is that individuals do not generally act until they have gained sufficient knowledge about an action and its consequences, have a positive attitude towards it, have talked to others about it, and until it feels right for them. These psychosocial factors directly influence behaviour and in turn can be influenced by strategic social and behaviour change interventions. The model also includes how changes in the skills and knowledge necessary to carry out a particular action and the environmental context can complement and reinforce changes in ideation. For malaria, important contextual factors may include malaria policy, access to prevention, diagnostic and treatment commodities, socioeconomic conditions, geographical location and housing structure.

The ideation model has recently been applied to understand the psychosocial determinants of malaria-related behaviours across settings, including insecticide-treated net ownership and use,^3^^–^^5^ care-seeking for fevers by caretakers of young children and administration of artemisinin-based combination therapies,^6^^,^^7^ and receiving preventive treatment with sulfadoxine–pyrimethamine during pregnancy.^8^ These studies showed a strong correlation between ideational factors and malaria-related behaviours. Studies have shown that social and behaviour change interventions that employ the ideation model can significantly improve message exposure, ideation and household coverage of insecticide-treated nets, among other behaviours.^7^

The same studies also suggest that malaria behaviours are often influenced by multiple psychosocial determinants simultaneously. For example, a study of the ideational factors associated with consistent use of bed nets in Nigeria identified the importance of factors, such as perceptions about severity and susceptibility, perceived self-efficacy to use and obtain nets, and perceived response-efficacy of bed nets.^6^ The importance of different factors can vary across contexts and across specific behaviours, highlighting the importance of locally available evidence to inform social and behaviour change programmes. For example, a three-country study of the correlates of care-seeking for children with fever found that significant correlates differed largely across countries.^9^ Malaria ideation and behaviours can also vary based on factors such as sex, socioeconomic status and rural versus urban location, underscoring the importance of measuring and controlling for these factors.^6^^–^^11^

## Improving malaria control 

Malaria social and behaviour change strategies and programmes are often designed in the absence of data on the psychosocial determinants of specific target behaviours, resulting in activities that fail to fully address the needs of individuals and communities. Consistently measuring behavioural determinants can help national malaria control programmes and partners understand which combination of individual and contextual factors are most likely to contribute to behaviour change in each context and effectively monitor progress.

An example of an evidence-based approach is the Malaria Behaviour Survey, developed by the Johns Hopkins Center for Communication Programs, with support from the United States President’s Malaria Initiative.^12^ This is a cross-sectional household survey that includes validated questions measuring a full range of malaria-related behaviours, general malaria and behaviour-specific ideational factors, contextual factors such as access to malaria interventions, and sociodemographic characteristics. The survey uses a standardized method to produce data to inform malaria social and behaviour change interventions, allow for comparison across contexts and provide a framework for monitoring progress.^12^

A malaria behaviour survey toolkit, including standard questionnaires, is available.^12^ To date, the survey has been implemented in or planned for 10 countries in partnership with national malaria control programmes, the United States President’s Malaria Initiative, and in some countries, the Global Fund to Fight AIDS, Tuberculosis and Malaria. The results will directly inform social and behaviour change strategies in those countries.

Major progress has been achieved in the fight against malaria; however, to make further gains, the user perspective must be better addressed to ensure that a critical mass of families and communities adopt malaria-related behaviours. A systematic approach for measuring and analysing relevant human behavioural factors can help national malaria control programmes and partners design more effective combinations of interventions across different contexts. Improving understanding of human behaviour and elevating the role of evidence-based, theory-informed social and behaviour change is an important and necessary step towards increasing the impact of malaria control efforts globally. Proven approaches, such as use of the ideation model to design and evaluate social and behaviour change interventions, provide examples of how to effectively transform the theory of social and behaviour change into practical interventions to alleviate the malaria burden and accelerate the path towards its elimination.
