# Corrigendum

**DOI:** 10.2471/BLT.21.101221

**Published:** 2021-12-01

In: Monroe A, Olapeju B, Moore S, Hunter G, Payne Merritt A, et al. Improving malaria control by understanding human behaviour. Bull World Health Organ. 2021 Nov 1;99(11):837–839, 

On page 839, reference 3 should read as follows: 

Kincaid DL, Storey JD, Figueroa ME, Underwood CR. Communication, ideation, and contraceptive use: the relationships observed in five countries. In: World Congress on Communication for Development; 2006 Oct 25–27; Rome, Italy. Washington DC: World Bank; 2007. 

Bull World Health Organ. 2021 Nov 01;99(11):837–9. https://doi.org/10.2471/BLT.20.285369

